# Application of Microsatellite Markers in Conservation Genetics and Fisheries Management: Recent Advances in Population Structure Analysis and Conservation Strategies

**DOI:** 10.1155/2014/691759

**Published:** 2014-04-07

**Authors:** P. M. Abdul Muneer

**Affiliations:** ^1^National Bureau of Fish Genetic Resources (NBFGR) Cochin Unit, CMFRI Campus, Cochin, Kerala 682 018, India; ^2^JFK Medical Center, Edison, NJ 08820, USA

## Abstract

Microsatellites are the most popular and versatile genetic marker with myriads of applications in population genetics, conservation biology, and evolutionary biology. These are the arrays of DNA sequences, consisting of tandemly repeating mono-, di-, tri-, and tetranucleotide units, which are distributed throughout the genomes of most eukaryotic species. Microsatellites are codominant in nature, highly polymorphic, easily typed, and Mendelian inherited, all properties which make them very suitable for the study of population structure and pedigree analysis and capable of detecting differences among closely related species. PCR for microsatellites can be automated for identifying simple sequence repeat polymorphism. Small amount of blood samples or alcohol preserved tissue is adequate for analyzing them. Most of the microsatellites are noncoding, and therefore variations are independent of natural selection. These properties make microsatellites ideal genetic markers for conservation genetics and fisheries management. This review addresses the applications of microsatellite markers in conservation genetics and recent advances in population structure analysis in the context of fisheries management.

## 1. Introduction

Organisms are incessantly undergoing micro- and macroevolutionary processes both at molecular and organismal levels. In fact, the process of evolution starts at the molecular level, more precisely from a single base of the DNA molecule, and ends up in variations at the organismal level. Genes are the factors, which determine the phenotypic characters of any organism. Thus, the variations that happen to the genes in turn produce individuals, which are different either at the molecular level or at the organismal level. These individuals may form separate groups within the species itself and such groups are the fundamental genetic units of evolution. These intraspecific groups were called as “stocks” and fishery biologists started using these stocks as a basis to manage commercially important marine organisms. Shaklee et al. [1] defined a stock as “a panmictic population of related individuals within a single species that is genetically distinct from other such populations.” Therefore, in any management regime, identification of discrete stocks becomes a critical element [2, 3].

Genetic variation in populations became a subject of scientific enquiry in the late nineteenth century prior even to the rediscovery of Mendel's paper in 1900. Genetic variation, in the form of multiple alleles of many genes, exists in most natural populations. In most sexually reproducing populations, no two organisms (barring identical twins or other multiple identical births) can be expected to have the same genotype for all genes [4]. In 1990s, genetic markers became more popularized for the identification of stock structure and genetic variation in a population. The detection of genetic variation among individuals is a requirement in all applications of genetic markers in fisheries biology. A genetically inherited variant in which the genotype can be inferred from the phenotype during genetic screening is known as a genetic marker. The most common use of genetic markers in fisheries biology is to determine if samples from culture facilities or natural populations are genetically differentiated from each other. They are also used to identify different species in the event of taxonomic disputes and to detect genetic introgression in a species. The detection of genetic differentiation would imply that the source groups comprise different stocks [5] and should be treated as separate management units or stocks [6]. A common objective of molecular genetic analyses is to find diagnostic differences among presumed stocks in either nuclear allelic types or mtDNA haplotypes [7]. Polymorphic DNA markers can provide fisheries researchers with new insights into the behavior, ecology and genetic structure of fish populations, levels of inbreeding, disassortative mating success of alternative reproductive strategies and life histories, and the intensity of natural and sexual selection [8]. Microsatellites are one of the best suitable genetic markers for analyzing pedigree, population structure, genome variation, evolutionary process, and fingerprinting purposes.

Genetic markers are basically two types—protein and DNA (molecular). In the beginning of 1960s, the proteins such as haemoglobin and transferrin were involved in all studies. In protein markers, allozyme markers are very popular and most of the genetic variation studies have been conducted based on this marker [9–14]. Molecular markers can be categorized into two classes, nuclear DNA and mitochondrial DNA (mtDNA) markers, based on their transmission and evolutionary dynamics [15]. Nuclear DNA markers such as random amplified polymorphic DNA (RAPDs), amplified fragment length polymorphisms (AFLPs), variable number of tandem repeats loci (VNTRs: minisatellites, microsatellites), and single nucleotide polymorphisms (SNPs) are biparently inherited. Mitochondrial DNA markers are maternally inherited, exhibit high rates of mutation, and are nonrecombining such that they have one-quarter the genetic effective population size (Ne) of nuclear markers [8]. Using restriction enzymes mtDNA sequence can be cut at specific sites to generate restriction fragment length polymorphisms (RFLPs) or sequence analysis of different genes of mtDNA can be used to detect phylogenetic relationships, undertake pedigree analysis, and assess population differentiation in many species.

Detection of polymorphisms at the nucleotide sequence level represents a new area for genetic studies, especially as technologies become available, which allow routine application with relative ease and low cost. From the 1990s an increasing number of studies have been published making use of random parts of a genome. With the advent of thermocyclers, the amplification of small fragment of DNA through polymerase chain reaction (PCR) gained popularity. The PCR technique was discovered in 1985 and the development of DNA amplification using the PCR technique has opened the possibility of examining genetic changes in fish populations over the past 100 years or more using archive materials such as scales [8]. The advent of PCR coupled with automated DNA sequencers made feasible major technological innovations such as minisatellite variant repeat mapping [16] and assessment of the variations at microsatellite loci [17]. The PCR based techniques have the added attraction of requiring only extremely small amounts of DNA that has led to wide usage of this technique in aquaculture and fisheries. In this review, we discuss the application of the most prevalent genetic marker, microsatellites, in population genetic structure and its usefulness in conservation of fish fauna.

## 2. Microsatellites Markers

Recently, attention has turned to another type of genetic variation that of differences in the number of repeated copies of a segment of DNA. These sequences can be classified based on decreasing sizes into satellites, minisatellites, and microsatellites [13]. Satellites consist of units of several thousand base pairs, repeated thousands or millions of times. Minisatellites consist of DNA sequences of some 9–100 bp in length that are repeated from 2 to several 100 times at a locus. Minisatellites discovered in human insulin gene loci with repeat unit lengths between 10 and 64 bp were also referred to as “variable number of tandem repeats” (VNTRs) DNA [18]. Microsatellites have a unique length of 1–6 bp repeated up to about 100 times at each locus [19]. They are also called as “simple sequence repeat” (SSR) by Tautz [13] or “short tandem repeat” (STR) DNA by Edwards et al. [20]. Jeffreys et al. [21] and Weber [22] opined that length variations in tandemly arrayed repetitive DNA in mini- and microsatellites are usually due to an increase or decrease in repeat unit copy numbers. Differences in repeat numbers represent the base for most DNA profiling techniques used today. Later, only microsatellites became very common in population genetics studies.

Microsatellites are short tandemly arrayed di-, tri-, or tetranucleotide repeat sequences with repeat size of 1–6 bp repeated several times flanked by regions of nonrepetitive unique DNA sequences [13]. Polymorphism at microsatellite loci was first demonstrated by Tautz [13] and Weber and May [17]. Alleles at microsatellite loci can be amplified by the polymerase chain reaction [23] from small samples of genomic DNA and the alleles separated and accurately sized on a polyacrylamide gel as one or two bands and they are used for quantifying genetic variations within and between populations of species [24]. The very high levels of variability associated with microsatellites, the speed of processing, and the potential to isolate large number of loci provide a marker system capable of detecting differences among closely related populations. Microsatellites that have been largely utilized for population studies are single locus ones in which both the alleles in a heterozygote show codominant expression [25]. Individual alleles at a locus differ in the number of tandem repeats and as such can be accurately differentiated on the basis of electrophoresis (usually PAGE) according to their size. Different alleles at a locus are characterized by different number of repeat units. They give the same kind of information as allozymes: distinguishable loci with codominant alleles, but they are generally neutral and more variable than allozymes [26]. Like allozymes, microsatellites alleles are inherited in a Mendelian fashion [27]. Moreover, the alleles can be scored consistently and compared unambiguously, even across different gels. An additional advantage is that they allow the use of minute or degraded DNA [26].

**Figure 1:**
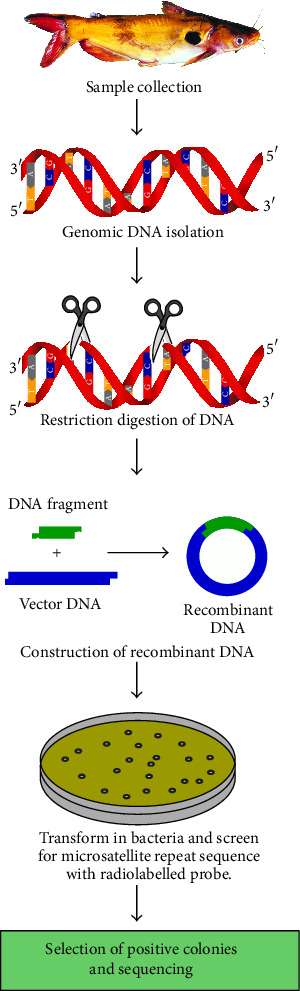
Schematic representation of traditional method of development of species specific microsatellite markers.

**Figure 2:**
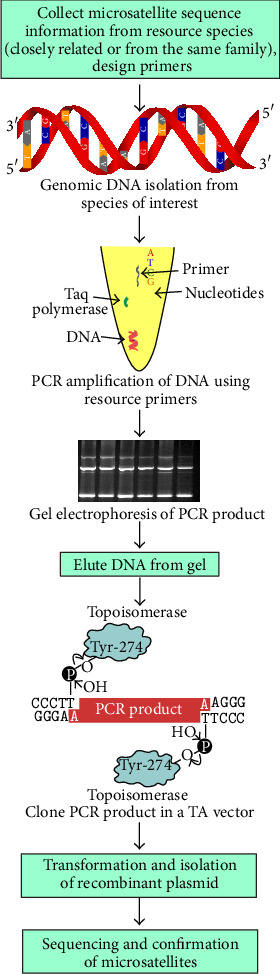
Schematic representation of development of microsatellite markers by cross-species amplification.

Generally, microsatellite loci are abundant and distributed throughout the eukaryotic genome [28] and each locus is characterized by known DNA sequence. These sequences consist of both unique DNA (which defines the locus) and repetitive DNA motifs (which may be shared among loci). The repetitive elements consist of tandem reiterations of simple sequence repeats (SSRs) and are typically composed of two to four nucleotides such as (AC)*n* or (GATA)*n* where* n* lies between 5 and 50 [29]. Within vertebrates, the dinucleotide repeats -GT and CA- are believed to be the most common microsatellites [30]. Study of single locus microsatellites requires specific primers flanking the repeat units, whose sequences can be derived from (i) genomic DNA libraries or (ii) from available sequences in the gene banks (Figures 1 and 2). These two methods are generally used for the development of microsatellite markers. The second method is extensively described in the coming section. In a review, Zane et al. [31] showed several methods of development of microsatellite markers.

## 3. Advantages of Microsatellite Markers

The major advantages of microsatellite markers are codominant transmission (the heterozygotes can be distinguished from homozygotes), locus-specific in nature, highly polymorphic and hypervariable, high information content and providing considerable pattern, relative abundance with uniform genome coverage, higher mutation rate than standard, and easy to sample preparation. Advantages of microsatellites such as short size range, uninterrupted stretches of identical repeat units, high proportion of polymorphisms, insights gained in understanding the mutational process which helps in developing statistical procedures for interpopulation comparisons, their abundance in fish genomes, the availability of methodologies for cloning of microsatellites have all resulted in their abundant use in fisheries research. Tetranucleotide microsatellites are also very useful for paternity and forensic investigations in humans. The advantageous properties of microsatellites have led to modern developments such as digital storage and automated detection and scoring systems such as automated DNA sequences and fluorescent-imaging devices [27]. Disadvantages of microsatellites include the appearance of shadow or stutter bands, presence of null alleles (existing alleles that are not observed using standard assays), homoplasy, and too many alleles at certain loci that would demand very high sample size for analysis [32]. Also, microsatellite flanking regions (MFRs) sometimes contain length mutations which may produce identical length variants that could compromise microsatellite population level studies (and comparisons of levels of variation across species for homologous loci) and phylogenetic inferences as these length variants in the flanking regions can potentially minimize allele length variation in the repeat region [30].

## 4. Application of Microsatellites in Population Structure Analysis in Fisheries and Aquaculture

**Figure 3:**
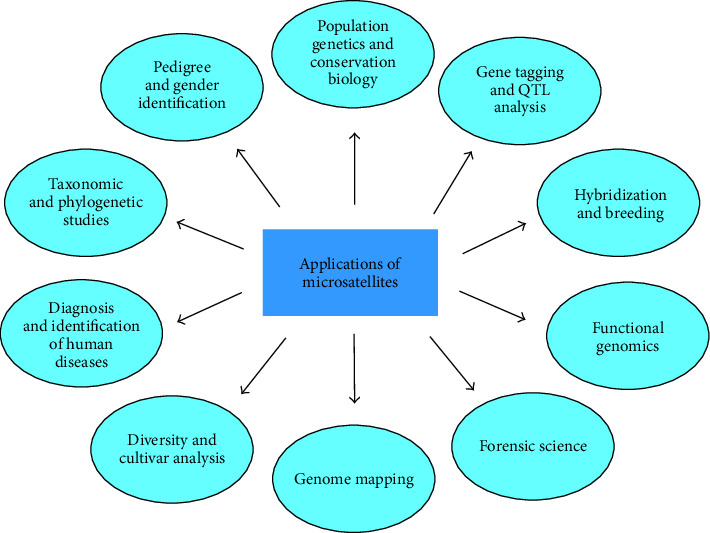
Applications of microsatellite markers in different areas.

The high variability, ease, and accuracy of assaying microsatellites make them the marker of choice for high-resolution population analysis [33]. Microsatellites with only a few alleles are well suited for population genetic studies, while the more variable loci are ideal for genome mapping and pedigree analysis and the fixed or less polymorphic microsatellite loci are used to resolve taxonomic ambiguity in different taxa [5]. Highly polymorphic microsatellite markers have great potential utility as genetic tags for use in aquaculture and fisheries biology. They are powerful DNA markers for quantifying genetic variations within and between populations of species [25]. They may prove particularly valuable for stock discrimination and population genetics due to the high level of polymorphism compared with conventional allozyme markers [34, 35]. Microsatellite DNA markers are among the most likely to conform to the assumption of neutrality and have proven to be powerful in differentiating geographically isolated populations and sibling species and subspecies [30]. The qualities of microsatellites make them very useful as genetic markers for studies of population differentiation and stock identification [35, 36], in kinship and parentage exclusion [37, 38] and in genome mapping [39]. Microsatellites are also being used as genetic markers for identification of population structure, genome mapping, pedigree analysis, and to resolve taxonomic ambiguities in many other animals besides fishes [40–49]. The broad areas of applications of microsatellite markers are depicted in Figure 3.

Various authors have reported microsatellite polymorphisms and sequences in some marine and freshwater fish species for population genetic analysis [25, 34, 50–55]. The development of polymorphic microsatellite markers to determine the population structure of the Patagonian toothfish,* Dissostichus eleginoides*, has been reported by [56, 57]. Similarly, Appleyard et al. [58] examined seven microsatellite loci in the same species of Patagonian toothfish from three locations in the Southern Ocean. Microsatellite polymorphisms have been used to provide evidence that the cod in the northwestern Atlantic belongs to genetically distinguishable populations and that genetic differences exist between the northwestern and southeastern cod populations [59]. Recently, Larsen et al. [60] showed differences in salinity tolerance and its gene expression in two populations of Atlantic cod (*Gadus morhua*). Drinan et al. [61] reported 20 microsatellites for determining the patterns of population genetic variation in westlope cutthroat trout,* Oncorhynchus clarkia* lewisii in 25 populations from four rivers. Davies et al. [62] identified 12 microsatellite loci in tuna species of genus* Thunnus* and investigated genetic polymorphism at these loci in North Atlantic and Mediterranean Sea populations. In a cichlid,* Eretmodus cyanostictus*, Taylor et al. [63] determined four polymorphic microsatellite loci for studying nine populations in Lake Tanganyika. In another study, recently, 7 polymorphic microsatellite markers were identified in snakehead murrel,* Channa striata,* from Malaysia [64]. Similarly, several authors reported population genetic structure of different species of catfish; few of them are in the farmed catfish from Tamaulipas, Mexico [65]; in neotropical catfish [66]; in* Pseudoplatystoma reticulatum* [67]. O'Connell et al. [24] reported the investigation of five highly variable microsatellite loci for population structure in Pacific herring,* Clupea pallasi,* collected from 6 sites in Kodiak Island. Similarly, many others have reported studies of polymorphic microsatellite loci to evaluate population structure of different fish species. Thus microsatellite markers have wide range of applications in population genetics and fisheries management.

Salzburger et al. [68] reported a case of introgressive hybridization between an ancient and genetically distinct cichlid species in Lake Tanganyika that led to the recognition of a new species. This is evidenced by the analysis of flanking regions of the single copy nuclear DNA locus (Tmo M27) and by studying the parental lineages in six other microsatellite loci. Leclerc et al. [69] had cloned and characterized a highly repetitive DNA sequence from the genome of the North American* Morone saxatilis* that was used to distinguish the four other species. Neff et al. [70] described 10 microsatellite loci from blue gill (*Lepomis macrochirus*) and discussed their evolution within the family Centrarchidae. Kellogg et al.[71] applied microsatellite-fingerprinting approach to address questions about paternity in cichlids. The usefulness of microsatellite markers for genetic mapping was determined in* Oreochromis niloticus* by Lee and Kocher [72], while Brooker et al. [73] reported the difference in organization of microsatellite between mammals and cold water teleost fishes. DeWoody and Avise [29] reported microsatellite variation in marine, fresh water, and anadromous fishes compared with other animals. Microsatellite DNA variation was used for population structure in* Oncorhynchus kisutch* [74], Atlantic salmon [75], and in Brown Trout,* Salmo trutta* [76]. Microsatellite markers have been studied in a few cyprinids also. Naish and Skibinski [77] studied tetranucleotide (TCTA) repeat sequences in Indian major carp,* Catla catla,* as potential DNA markers for stock identification. Gopalakrishnan et al. [51] and Das et al. [78] carried out characterization of dinucleotide microsatellite repeats in* Labeo rohita*.

## 5. Development of Microsatellite Markers by Cross-Species Amplification

Although microsatellite DNA analysis via PCR is an ideal technique for answering many population genetic questions, the development of species-specific primers for PCR amplification of alleles can be expensive and time-consuming, as it involves construction of genomic libraries, screening of clones with microsatellite sequences, and designing microsatellite primers. However, there are reports which point to the fact that flanking sequences of some microsatellite loci are conserved among related taxa so that primers developed for one species can be used to amplify homologous loci in related species. The method of microsatellite markers development by cross-species amplification is shown in Figure 2. The conservation of flanking regions of microsatellite sequences among closely related species has been reported by a number of groups [79–82]. Such an approach can circumvent extensive preliminary work necessary to develop PCR primers for individual loci that continues to stand in the way of quick and widespread application of single locus microsatellite markers. Thus, by using heterologous PCR primers the cost of developing similar markers in related species can be significantly reduced.

Schlotterer et al. [83] found that homologous loci can be amplified from a diverse range of toothed (Odontoceti) and baleen (Mysticeti) whales with estimated divergence times of 35–40 million years. Moore et al. [84] found that microsatellites flanking regions were conserved across species as diverse as primates, artiodactyls, and rodents. Microsatellite primers developed from foxtail millet (*Setaria italica* L) were used in studies of other millets and nonmillets species [85]. Similarly, primers developed for passerine birds were used in studies of a variety a of other bird species [86].

A number of attempts have been made to study the cross-species amplification of microsatellite loci in fishes. Recently, Gupta et al. [87] developed polymorphic microsatellite markers in featherback,* Notopterus notopterus,* by cross-species amplification of primers developed in 3 fish species of families notopteridae and osteoglossidae. Polymerase chain reaction (PCR) microsatellite multiplex assays were established for genetic studies of the population structure, hybridization and conservation status of European whitefish,* Coregonus lavaretus L.,* and cross-species amplification and rearrangement of the same loci analyzed in* C. albula L *[88]. Dubut et al. [89] have developed five multiplex PCR sets optimized to analyze 41 cyprinid-specific polymorphic microsatellite loci (including 10 novel loci isolated from* Chondrostoma nasus, Chondrostoma toxostoma, *and* Leuciscus leuciscus*) for the individuals from other different European cyprinid species.

**Figure 4:**
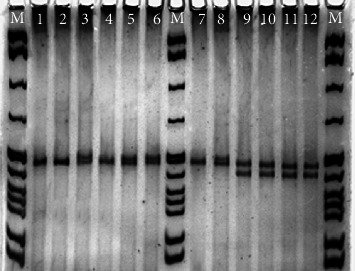
Cross-species amplification of microsatellite markers for the population genetic structure from three river systems in* Horabagrus brachysoma* (yellow catfish) from the primer (Cga06) developed in* Clarias gariepinus* (African catfish). The data of this figure has been published by Abdul Muneer et al. [25]. M molecular weight marker (*pBR322 with MspI cut*).

We have developed several microsatellite markers in different fresh water species by cross-species amplification. In* Horabagrus brachysoma*, an endangered yellow catfish, we have developed eight microsatellite markers from other catfish of order Siluriformes [25, 52].  Figure 4 shows the cross-species amplification microsatellites in* Horabagrus brachysoma* from the primer developed in African catfish,* Clarias gariepinus* [25]. In addition, we developed microsatellite markers for differentiating two species of endangered catfish,* Horabagrus,* by using the primers of Siluriformes and Osteoglossiformes [79]. May et al. [90] reported microsatellite genetic variation through cross-species amplification in sturgeons* Acipenser* and* Scaphirhynchus*. Takagi et al. [91] reported that microsatellite primers isolated from one tuna might be used to amplify microsatellite loci in other tuna species especially those of the genus* Thunnus*. Microsatellites from rainbow trout* Oncorhynchus mykiss* have been used for the genetic study of salmonids [75, 92]. Heterologous primers have been used to characterize bull trout by using three sets of primers from sockeye salmon, rainbow trout, and brook trout [93], for several* Salvelinus *species using primers of* Salvelinus fontinalis, *for Brook charr [94] and* Oreochromis shiranus *and* O. shiranus chilwae* by using primers of Nile tilapia [95]. The cross-species amplification of 32* Oreochromis niloticus* microsatellite markers from 15 different African cichlid species was successfully tested and analyzed [96]. There are some reports in which the flanking sequences are conserved between families of the same order. Primers of stickleback and cod have been used in* Merlangius merlangus* (Gadidae) [97], that of rainbow trout (Family: Salmonidae) in whitefish,* Coregonus nasus *[98], and primers of goldfish,* Carassius auratus,* in nine species of cyprinids [99]. Yue et al. [100] developed 15 polymorphic microsatellite loci in silver crucian carp* Carassius auratus gibelio* and reported eleven out of 15 primer pairs cross-amplified in the genome of common carp (*Cyprinus carpio*). Zardoya et al. [30] through a classical study demonstrated that microsatellite flanking regions (MFRs) contain reliable phylogenic information and they were able to recover with considerable confidence the phylogenetic relationship within family Cichlidae and other families of the suborder Labroidei from different parts of the world. Mohindra et al. [32] have carried out cross-species amplification of* C*.* catla* G1 primer in* Catla catla *from Gobind Sagar,* Labeo dero, L. dyocheilus, L. rohita,* and* Morulius calbasu*, and sequenced the loci in these species. Das et al. [78] also carried out characterization of dinucleotide microsatellite repeats in* Labeo rohita*. Recently, we successfully developed polymorphic microsatellite markers for* Gonoproktopterus curmuca* through cross-species amplification of primers from other cyprinid fishes [101, 102]. The development of 59 polymorphic microsatellite markers in silver crucian carp (*Carassius auratus gibelio*) and its successful cross-species amplification have been reported in crucian carp (*Carassius auratus*) [103].

Microsatellites have become the genetic markers of choice for studies of population differentiation and parentage determination. However, several microsatellite loci are required for such studies in order to obtain an appropriate amount of genetic polymorphism [9, 104]. Fortunately, genotypic data collection has become efficient through the development of automated DNA sizing technology using fluorescent-labelled DNA and coamplification of multiple loci in a single PCR [24, 105].

## 6. Importance of Microsatellite Markers in Conservation and Fisheries Management

The microsatellite markers study generate important information on the genetic variation and stock structure of fish species and it is a significant step towards realizing the goal of management of fishery and conservation of the species in their natural populations. The differentiation of a species into genetically distinct populations is a fundamental part of the process of evolution and it depends upon physical and biological forces such as migration, selection, genetic drift, and geographic barriers. Endangered species will have small and/or declining populations, so inbreeding and loss of genetic diversity are unavoidable in them. Since inbreeding reduces reproduction and survival rates and loss of genetic diversity reduces the ability of populations to evolve to cope with environmental changes, Frankham [106] suggested that these genetic factors would contribute to extinction risk especially in small populations of threatened species. With the loss of a population/genetic stock, a species also loses its members adapted and evolved to survive in particular habitat. Hence, conservation and fishery management strategy need to be stock-specific.

In population genetic analysis, low genetic variability (heterozygote deficiency and deviation from Hardy-Weinberg equilibrium) coupled with inbreeding (positive value of *F*_IS_) show consequence of genetic bottleneck, resulting from overexploitation and habitat [107]. As these factors would lead to a reduction in reproductive fitness [108], efforts to increase the genetic diversity of the fish species should be given high priority for conservation of the species, based on genetic principles as mentioned below.

- The effective population size (Ne) should be maintained as large as possible to maximize the contribution of a large number of adults for reproduction so as to maintain natural genetic variability.
- The causative factors that reduce the effective population size such as overexploitation should be controlled at the earliest.
- No artificial gene flow between distinct stocks should be created by means of haphazard stocking and rehabilitation programs.
- The rehabilitation strategy should also include means (screening the population, using genetic markers) to monitor impact of such program.

To attain these objectives, it is essential (i) to protect the populations and habitat against anthropogenic stress and (ii) enhance the population through propagation assisted stock-specific rehabilitation programs:

- regulation of human activities either self-imposed (public understanding and awareness through education) or state imposed (formulation and implementation of suitable laws),
- imposing ban on fishing practices particularly during breeding seasons,
- stock assessment of different rivers and imposing quota systems for maintaining the population size,
- banning the sale of undersized specimens,
- restricting the fishing gear for not catching small and immature fish species and preventing the use of explosives and chemicals for fishing,
- maintaining minimum water level in the rivers (in case there are dams and weirs) and declaring certain stretches of rivers as sanctuaries.

The natural populations of the endangered species can be enhanced by “supportive breeding.” In this program, a fraction of the wild parents are bred in captivity and the progeny are released in natural waters.

1. Brood stock of fish species collected from different rivers must be tagged and maintained in separate ponds in the holding facility.
2. Effective breeding population size and sex ratio should not be restricted. To achieve this, collection of different size/year classes at different time intervals is to be preferred over the same size/year class.
3. Use of cryopreserved milt, collected from different males and pooled, would be useful for increasing the effective population size and recovery of endangered populations of fish species. In comparison to the captive breeding program, the gene banking through sperm cryopreservation is relatively cheaper, easy to maintain, and less prone to risk due to system failure or mortality due to diseases. Therefore, it should serve as a useful adjunct to the captive breeding program.
4. Different genetic stocks should be bred separately and ranched in the same rivers from where they are collected.
5. Stretches of rivers harbouring resident population or that can serve as a potential sanctuary, may be selected for ranching of fish populations.
6. Assessing the impact of ranching through monitoring the parameters like catch per unit effort/area through experimental fishing should be done.
7. Changes in genetic variation, that is, allele frequencies, especially the occurrence of rare alleles over a course of time [19, 24] should be done. It will be useful to keep base genetic profile of representative samples of fish stocked in the holding facility and those used for ranching. Microsatellite markers and the baseline data generated in this study can be helpful in further assessing the impact of genetic variation.

## 7. Conclusion

Microsatellites are very powerful genetic markers for identifying fish stock structure and pedigree analysis and to study the genetic variation of closely related species. Microsatellite markers analysis provides essential information for formulating meaningful conservation strategies for fisheries and aquaculture management. This along with the other technologies like captive breeding and sperm cryopreservation can be integrated into a package for conserving genetic diversity and rehabilitation of the natural populations of fish species.
